# The Mechanism of Action of Ginkgolic Acid (15:1) against Gram-Positive Bacteria Involves Cross Talk with Iron Homeostasis

**DOI:** 10.1128/spectrum.00991-21

**Published:** 2022-01-12

**Authors:** Zewen Wen, Yuxi Zhao, Zhengyang Gong, Yuanyuan Tang, Yanpeng Xiong, Junwen Chen, Chengchun Chen, Yufang Zhang, Shanghong Liu, Jinxin Zheng, Duoyun Li, Qiwen Deng, Zhijian Yu

**Affiliations:** a Department of Infectious Diseases and Shenzhen Key Laboratory for Endogenous Infections, Huazhong University of Science and Technology Union Shenzhen Hospital, Shenzhen, China; b Guangdong Key Laboratory for Biomedical Measurements and Ultrasound Imaging, School of Biomedical Engineering, Shenzhen University Health Science Center, Shenzhen, China; c Shenzhen College of International Education, Shenzhen, China; Shenzhen Bay Laboratory

**Keywords:** ginkgolic acid (15:1), *E. faecalis*, ferric uptake regulator, Fur, antibacterial mechanism, iron homeostasis

## Abstract

With the increasing reports of community-acquired and nosocomial infection caused by multidrug-resistant Gram-positive pathogens, there is an urgent need to develop new antimicrobial agents with novel antibacterial mechanisms. Here, we investigated the antibacterial activity of the natural product ginkgolic acid (GA) (15:1), derived from Ginkgo biloba, and its potential mode of action against the Gram-positive bacteria Enterococcus faecalis and Staphylococcus aureus. The MIC values of GA (15:1) against clinical E. faecalis and S. aureus isolates from China were ≤4 and ≤8 μg/mL, respectively, from our test results. Moreover, GA (15:1) displayed high efficiency in biofilm formation inhibition and bactericidal activity against E. faecalis and S. aureus. During its inhibition of the planktonic bacteria, the antibacterial activity of GA (15:1) was significantly improved under the condition of abolishing iron homeostasis. When iron homeostasis was abolished, inhibition of planktonic bacteria by GA (15:1) was significantly improved. This phenomenon can be interpreted as showing that iron homeostasis disruption facilitated the disruption of the functions of ribosome and protein synthesis by GA (15:1), resulting in inhibition of bacterial growth and cell death. Genetic mutation of ferric uptake regulator (Fur) led to GA (15:1) tolerance in *in vitro*-induced resistant derivatives, while overexpression of Fur led to increased GA (15:1) susceptibility. Additionally, GA (15:1) significantly decreased the bacterial loads of S. aureus strain USA300 in the lung tissues of mice in a pneumonic murine model. Conclusively, this study revealed an antimicrobial mechanism of GA (15:1) involving cross talk with iron homeostasis against Gram-positive pathogens. In the future, the natural product GA (15:1) might be applied to combat infections caused by Gram-positive pathogens.

**IMPORTANCE** The increasing emergence of infectious diseases associated with multidrug-resistant Gram-positive pathogens has raised the urgent need to develop novel antibiotics. GA (15:1) is a natural product derived from Ginkgo biloba and possesses a wide range of bioactivities, including antimicrobial activity. However, its antibacterial mechanisms remain unclear. Our current study found that the function of ferric uptake regulator (Fur) was highly correlated with the antimicrobial activity of GA (15:1) against E. faecalis and that the antibacterial activity of GA (15:1) could be strengthened by the disruption of iron homeostasis. This study provided important insight into the mode of action of GA (15:1) against Gram-positive bacteria and suggested that GA (15:1) holds the potential to be an antimicrobial treatment option for infection caused by multidrug-resistant Gram-positive pathogens.

## INTRODUCTION

Gram-positive pathogens are the predominant cause of nosocomial and community-acquired infections (1, 2). The increasing emergence of infectious diseases associated with multidrug-resistant Staphylococcus aureus and *Enterococcus* strains, such as methicillin-resistant S. aureus (MRSA), linezolid-resistant enterococci (LRE), and vancomycin-resistant enterococci (VRE), has posed severe clinical challenges (3). Multidrug-resistant S. aureus and enterococcal infections are usually accompanied by increased length of stay, extra treatment cost, and high mortality (4, 5). Biofilm formation is another major factor that often contributes to clinical treatment failure, as biofilms cause bacterial cells to have high resistance to antibiotics, which often results in intractable infectious diseases (6). Multiple studies have shown that the bacterial cells embedded in a mature biofilm can tolerate antibiotics at concentrations 10 to 1,000 times higher than the concentrations that are effective under planktonic growth conditions (7). The increasing prevalence of multiple drug-resistant bacterial strains and their biofilm-forming abilities strengthen the current need to develop novel and effective antibiotics to treat bacterial infections.

A promising alternative to developing new antibacterial agents is to investigate the antibacterial effects of natural plant-derived active substances (8). As a rare, unique species in China, Ginkgo biloba L. is a famous living fossil of the gymnosperms, which can be traced back to over 300 million years ago. The leaves of Ginkgo biloba were used in ancient Chinese medicine to treat lung and cardiovascular diseases. Ginkgo acid (ginkgolic acid [GA]) is a mixture of a series of bioactive substances that mainly exist in sarcotesta/seed coats and belong to the long-chain phenolic derivatives of sumac acid. GA is described as 13:0, 15:0, 15:1, 17:1, and 17:2 based on the differences of side-chain carbon atoms and double bonds. Current studies have revealed that the biological activities of GA include antitumor, anti-inflammatory, neuroprotective, and antianxiety activities, antimicrobial activities *in vitro* (9–13), and broad antiviral activities against herpes simplex virus 1 (HSV-1), HIV, Ebola virus (EBOV), influenza A virus (IAV), Epstein Barr virus (EBV), and Chikungunya, Mayaro, Una, and Zika viruses by blocking the fusion event (12, 14–16). In addition, GA (15:1) was identified as a dominant ingredient that contributed to the antimicrobial activity of *Ginkgo* extracts (17). However, the antibacterial mechanisms of GA (15:1) remain unclear and poorly investigated. It is crucial to determine the mechanisms of action when evaluating new antimicrobial agents for clinical application, which can help to predict potential toxicity effects and improve the structure of GA (15:1) to enhance its antibacterial activity.

It has been reported that GA (15:1) has a potential antibacterial effect *in vitro* (17, 18). However, this activity and the underlying mechanisms responsible for it remain unclear. First of all, our study aimed to assess the antibacterial and antibiofilm activities of GA (15:1) against clinical multidrug-resistant Enterococcus faecalis and S. aureus strains. Furthermore, the *in vivo* effect of GA (15:1) against S. aureus was evaluated in a murine model with S. aureus pneumonia. Moreover, the antibacterial mode of action of GA (15:1) was investigated by quantitative proteomics. Finally, *in vitro* induction of GA (15:1) resistance and whole-genome sequencing were conducted to explore the mechanism of resistance to GA (15:1). The findings presented highlight the potential therapeutic application of GA (15:1) for treating infections caused by multidrug-resistant Gram-positive pathogens.

## RESULTS

### *In vitro* antimicrobial activity of GA (15:1) against Gram-positive pathogens.

In order to evaluate the antibacterial activity of GA (15:1), the MICs of GA (15:1) were determined in 60 clinical E. faecalis strains and 58 clinical S. aureus strains from China, including 30 methicillin-susceptible S. aureus (MSSA) isolates and 28 MRSA isolates. The results of the MIC assays are shown in Table 1, indicating that this natural product showed robust antibacterial activity against all clinical strains tested, including 14 linezolid-intermediate, three linezolid-resistant, and one vancomycin-intermediate E. faecalis isolate. The range of GA (15:1) MICs against clinical E. faecalis isolates was from 2 μg/mL to 4 μg/mL and for clinical S. aureus isolates was from 2 μg/mL to 8 μg/mL.

**TABLE 1:** MIC distributions of GA (15:1) and commonly used antibiotics against E. faecalis and S. aureus

Organism, antibiotic	MIC breakpoint (μg/mL)	No. of strains^*a*^
E. faecalis		
Ampicillin	≤8	59
	≥16	1
Tetracycline	≤4	10
	8	2
	≥16	48
Ciprofloxacin	≤1	38
	2	7
	≥4	15
Nitrofurantoin	≤32	57
	64	2
	≥128	1
Linezolid	≤2	43
	4	14
	≥8	3
GA (15:1)	≤ 2	2
	4	58

S. aureus		
Erythromycin	≤4	6
	≥8	54
Tetracycline	≤4	15
	8	5
	≥16	40
Ciprofloxacin	≤1	38
	≥4	22
Nitrofurantoin	≤32	58
	64	1
	≥128	1
Rifampicin	≤1	51
	≥4	9
GA (15:1)	≤2	5
	4	39
	8	14

a E. faecalis, *n* = 60; S. aureus, *n* = 58 (MSSA = 30, MRSA = 28).

**FIG 1:**
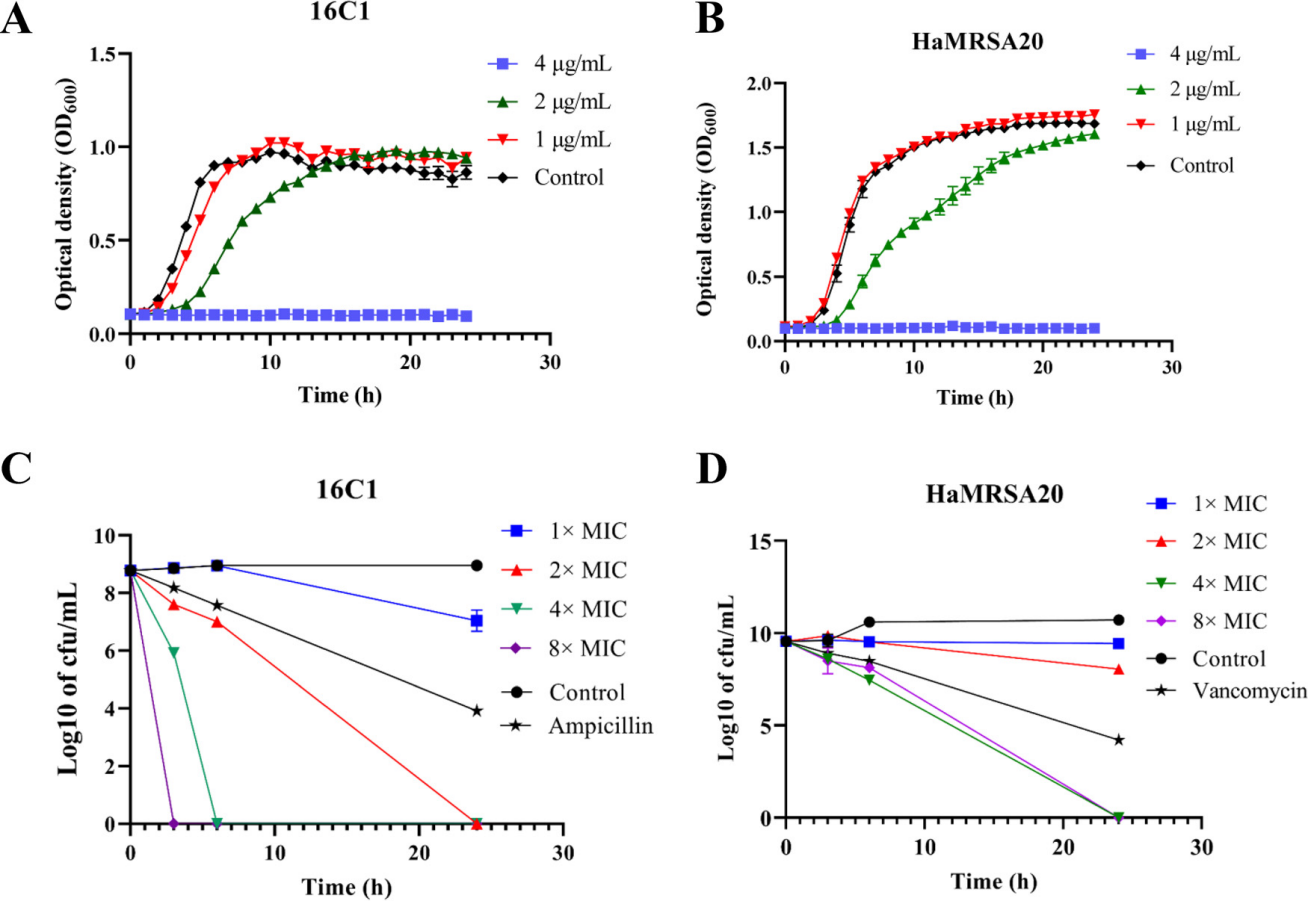
Bacterial growth curve and bactericidal effect analysis of GA (15:1) against E. faecalis (16C1) and MRSA HaMRSA20. (A, B) Impact of GA (15:1) at different concentrations (1/4×, 1/2×, and 1× MIC) on the bacterial growth of vancomycin-intermediate E. faecalis 16C1 and MRSA HaMRSA20 planktonic cells. (C, D) Time-kill assay of GA (15:1) with 1×, 2×, 4×, and 8× MIC against vancomycin-intermediate E. faecalis isolate 16C1 and MRSA HaMRSA20 cells at exponential phase. Data are presented as mean values ± standard deviations (SD). The control concentration (for both ampicillin and vancomycin) was 4× MIC.

Moreover, the inhibitory effects of GA (15:1) against clinical E. faecalis and S. aureus isolates were evaluated by automatic growth curve in an automatic growth instrument, demonstrating significant inhibition of two clinical E. faecalis and S. aureus isolates in the presence of 2 μg/mL and 4 μg/mL GA (15:1) (Fig. 1A and B).

Furthermore, the bactericidal activities of GA (15:1) at various concentrations (1× MIC, 2× MIC, 4× MIC, and 8× MIC) against planktonic bacterial cells (strains 16C1 and HaMRSA20) were addressed by time-kill assay. The results suggested that GA (15:1) could exert its bactericidal activity against E. faecalis and S. aureus strains in the exponential phase in a dose-dependent manner (Fig. 1C and D). E. faecalis cells treated with 4× or 8× MIC of GA (15:1) were completely exterminated after 3 h and 6 h, whereas the total bacterial counts of S. aureus treated with 4× or 8× MIC of GA (15:1) were decreased 3 log CFU/mL after 6 h, which was more effective than 4× MIC vancomycin. All the S. aureus cells were completely exterminated after 24 h. These results suggested that GA (15:1) displayed effective bactericidal activity against planktonic bacteria of clinical E. faecalis and S. aureus isolates at the exponential phase.

### Antibiofilm activity of GA (15:1) against S. aureus and E. faecalis.

**FIG 2:**
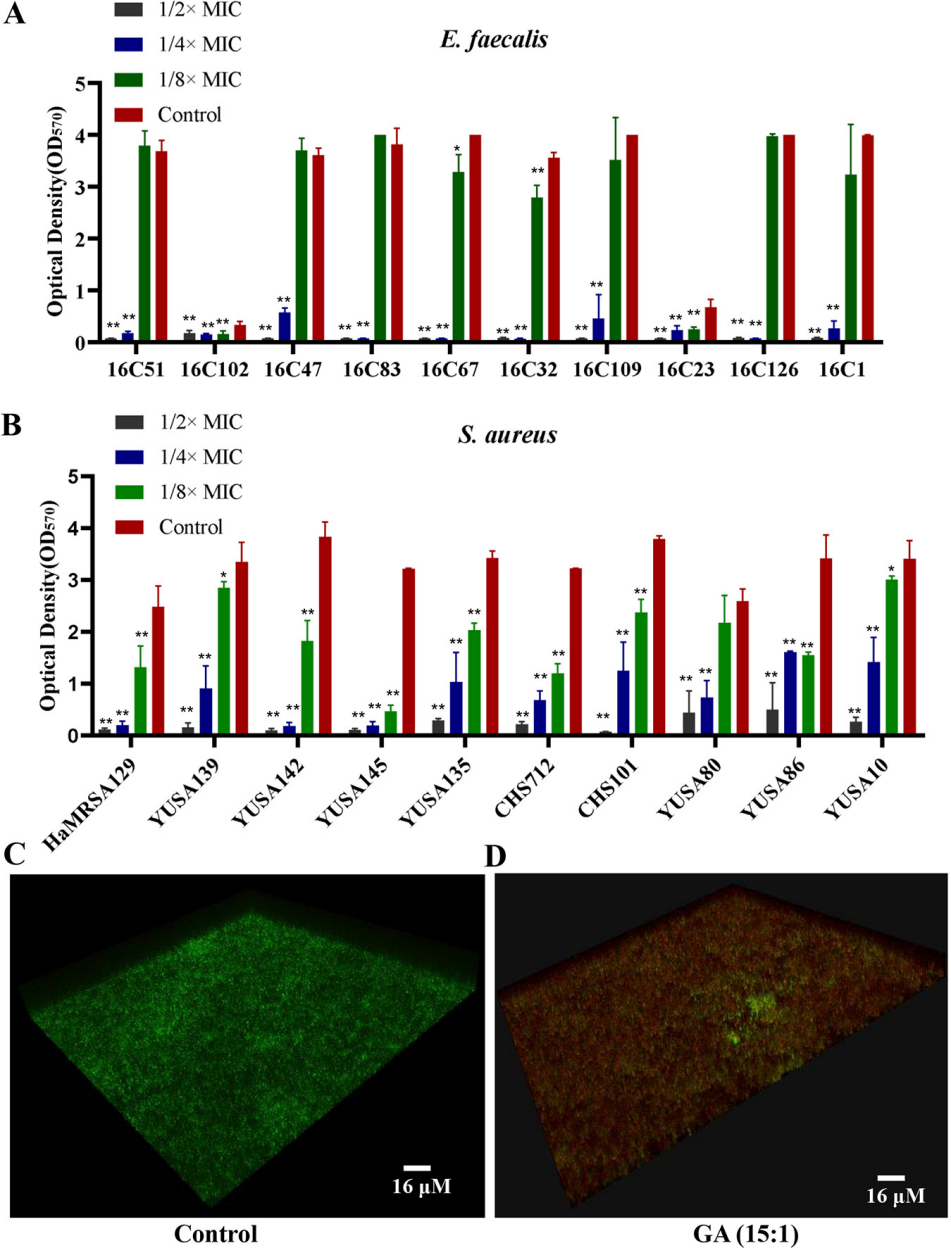
Antibiofilm activity of GA (15:1) against S. aureus and E. faecalis. (A, B) Significant inhibition of the biofilm formation of E. faecalis (A) and S. aureus (B) by GA (15:1) at different subinhibitory concentrations. The 10 E. faecalis strains, 5 MSSA strains, and 5 MRSA strains were treated with GA (15:1) at 1/2×, 1/4×, and 1/8× MIC for 24 h, and the biofilm formation was determined by crystal violet staining. The data presented are the average values from three independent experiments (means ± SD). *P* values are for comparison with the control: *, *P* < 0.05; **, *P* < 0.01 (Student’s *t* test). (C, D) Effect of GA (15:1) at 8× MIC against the viable cells embedded in mature biofilm of E. faecalis isolate 16C102. Bacterial cells were inoculated onto 96-well polystyrene microtiter plates for 24 h at 37°C until mature biofilms were formed. After being treated with GA (15:1) at 8× MIC or solvent control for another 24 h, the viability of the cells embedded in the mature biofilm was observed by confocal microscopy using LIVE/DEAD staining.

The antibiofilm effect of GA (15:1) against clinical S. aureus and E. faecalis isolates was assessed at different serial concentrations (1/8×, 1/4×, and 1/2× MIC) in 96-well plates. Quantitative assay of biofilm formation with crystal violet staining suggested that GA (15:1) had concentration-dependent inhibitory activity against biofilm formation of E. faecalis and S. aureus (Fig. 2A and B). Worthy of attention, GA (15:1) with a concentration of 1/8× MIC could cause significant decreases in the biofilm formation of E. faecalis strains 16C67, HaMRSA129, YUSA10, YUSA86, YUSA139, YUSA135, YUSA142, YUSA145, CHS101, and CHS712. In particular, GA (15:1) decreased the biofilm formation of all S. aureus and E. faecalis clinical isolates tested by at least 50% at 1/4× MIC, with unimpaired influence on the growth of planktonic cells. Moreover, the viable-cell count in the E. faecalis mature biofilm was assessed by confocal laser scanning microscopy (CLSM) using LIVE/DEAD staining. The results showed significantly reduced counts of live bacterial cells (stained green) and higher percentages of dead bacterial cells (stained red) in the presence of 8× MIC GA (15:1) in comparison to the results for the control (Fig. 2C and D), suggesting the capability of GA (15:1) for killing bacterial cells embedded in the biofilm.

### GA (15:1) is nonhemolytic and has limited cytotoxicity.

A hemolytic activity assay for GA (15:1) against fresh human red blood cells (RBCs) showed that it was nonhemolytic below a concentration of 32 μg/mL (Fig. S1A in the supplemental material). Cytotoxicity assay results also showed that no significant cytotoxicity was found in A549 and HUVEC cell lines incubated with 0 to 8 μg/mL of GA (15:1) for 24 h, while notable cytotoxicity was detected when the concentration of GA (15:1) was over 8 μg/mL (Fig. S1B).

### Intraperitoneal administration of GA (15:1) is safe and effective in the murine model with S. aureus pneumonia.

Safety studies using a single dose of 20 mg/kg of body weight or 50 mg/kg GA (15:1) with intraperitoneal injection showed no significant pathological changes in the lungs, liver, or kidneys of the treated groups compared to the results for the control group treated with phosphate-buffered saline PBS (Fig. S2A). Serum parameters were used to monitor the toxic effects of GA (15:1), suggesting that serum alanine transaminase (ALT) levels were significantly higher in the group treated with GA (15:1) at 50 mg/kg compared with the levels in the control group. No significant changes were observed between the two groups for other serum indicators, including aspartate transaminase (AST), blood urea nitrogen (BUN), and lactate dehydrogenase (LDH) release (Fig. S2B).

**FIG 3:**
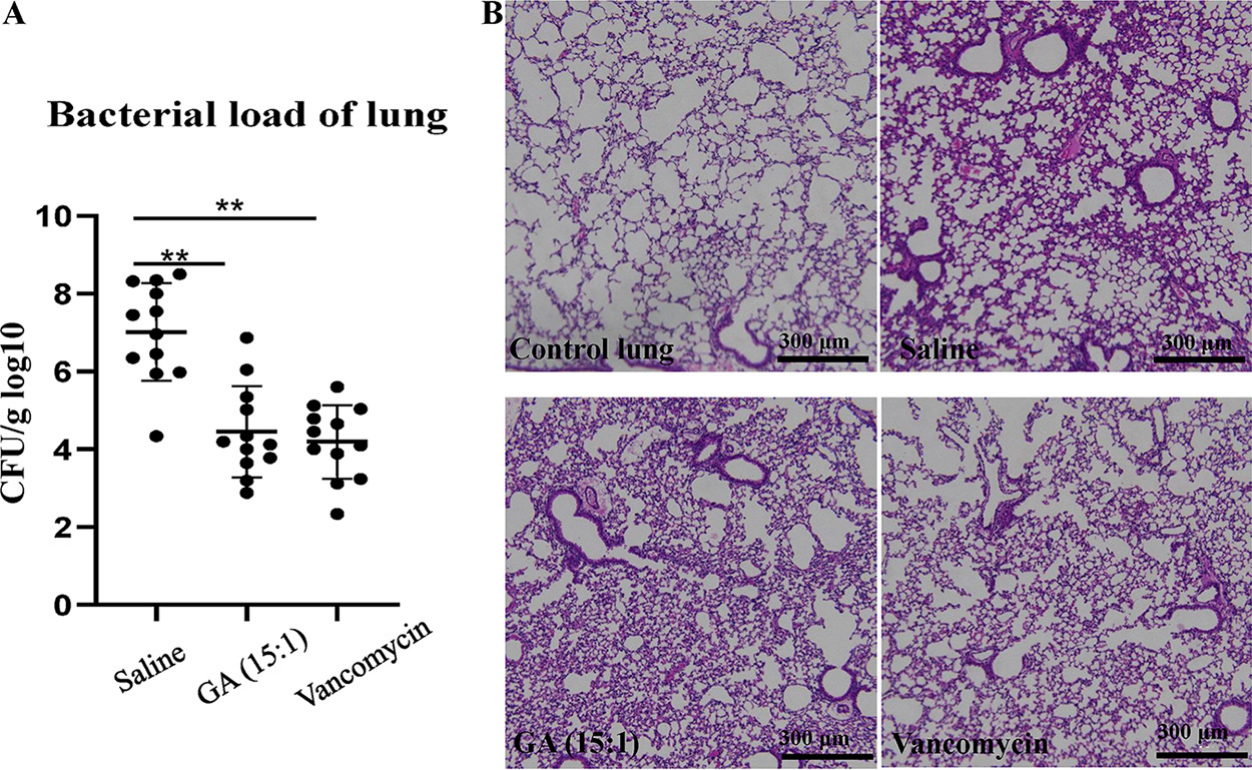
Protective effect of GA (15:1) against S. aureus USA300 pneumonia. Female BALB/c mice (*n* = 12/group) were challenged with S. aureus USA300 by nasal drip; GA (15:1) (25 mg/kg) or vancomycin (25 mg/kg) was administered to the mice by intraperitoneal injection 2 h before the bacterial challenge. (A) Bacterial burdens in the lungs were determined at 24 h postinfection. Data are presented as the mean values ± SD. **, *P* < 0.01 (Student’s *t* test). (B) Hematoxylin and eosin (H&E) staining (10×) of lung tissue showed that histopathological change (inflammatory cells) was significantly reduced after GA (15:1) treatment. Scale bars, 300 μm.

The murine model with S. aureus pneumonia was used to assay the *in vivo* antibacterial efficacy of GA (15:1) at the dose of 25 mg/kg. A bacterial burden of 2 × 10^8^ CFU of S. aureus USA300 was inoculated directly into the tracheas of the mice for the construction of the pneumonia infection model. GA (15:1) was intraperitoneally injected into the mice at a dose of 25 mg/kg 2 h before constructing the pneumonia infection model. Subsequently, the bacterial loads in the lung tissues of the mice were assessed at 24 h and 48 h after S. aureus infection (*n* = 12 per group). As shown by the results in Fig. 3, GA (15:1) treatment resulted in a marked decrease of the bacterial load, by 3 log, at 24 h after infection in comparison to the control, demonstrating that the bacterial growth was significantly inhibited by GA (15:1) in the murine pneumonia infection model. Furthermore, histopathological analysis showed that the inflammation of the lungs was significantly reduced in murine pneumonia after GA (15:1) treatment (Fig. 3B).

### Genetic mutations in GA (15:1)-induced nonsensitive isolates of E. faecalis.

**FIG 4:**
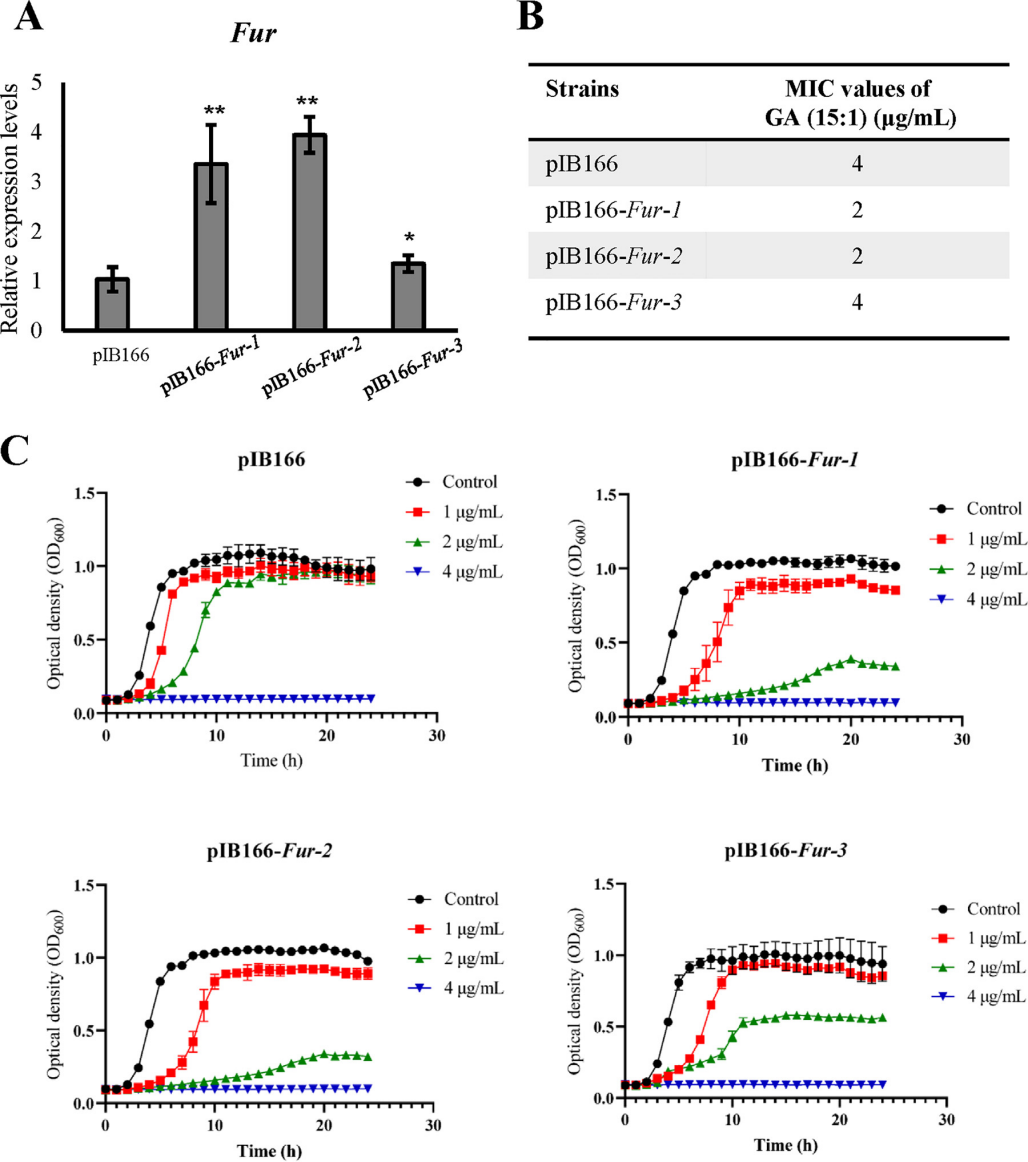
*fur* transcript levels and susceptibility analysis of GA (15:1) in three independent Fur overexpression transgenic isolates and pIB166 empty-vector control in E. faecalis strain OG1RF. (A) Three independent transgenic isolates of Fur overexpression strains (pIB166-*fur*-1, pIB166-*fur*-2, and pIB166-*fur*-1) and their empty-vector control in the exponential phase were used for total RNA extraction, and qRT-PCR was performed to determine the *fur* transcript levels. *gdh* was used as an internal control. Data are presented as the mean values ± SD. *, *P* < 0.05; **, *P* < 0.01 (Student’s *t* test). (B) MICs of GA (15:1) against Fur overexpression transgenic isolates and pIB166 empty-vector control in E. faecalis. (C) Bacterial growth curves of Fur overexpression E. faecalis strains after exposure to GA (15:1). Data are presented as mean values ± SD.

To explore the potential antibacterial mechanism of GA (15:1) against E. faecalis, two parental clinical E. faecalis isolates (16C51 and 16C166) were induced *in vitro* under GA (15:1) exposure. After 120 days of induction, two GA-resistant clones (16C51T1 and 16C166T1) were selected, and their GA MICs (15:1) were redetermined by the broth dilution method, showing a significant, 4-fold elevation. The genetic variations were then determined by comparing the whole-genome sequencing between the two parental sensitive isolates and their resistant clones (serially subcultured in tryptone soy broth [TSB]). Overall, 10 genes with nonsynonymous or stop gain mutations in strain 16C51T1 and 24 genes with nonsynonymous or prevent gain mutations in strain 16C166T1 were found by whole-genome sequencing and further confirmed by Sanger sequencing (Table S1). Interestingly, the ferric uptake regulator (Fur)-encoding gene, *EF1525*, and the YhgE/phage infection protein-encoding gene, GRB94_03620, presented in both nonsensitive isolates (Table S1). Thus, to confirm the roles of these two candidate genes in GA (15:1) resistance, overexpression analysis of Fur and YhgE in E. faecalis was conducted, and the overexpression levels were examined by real-time PCR (Fig. 4A). The antimicrobial susceptibility tests revealed that Fur overexpression caused the MIC values of GA (15:1) to decrease by 2-fold in E. faecalis strains with high transcriptional levels of *fur* (Fig. 4B). In addition, comparison of growth curves also revealed that Fur overexpression strains were more sensitive to GA (15:1) than the empty vector strain (Fig. 4C). These data indicated that Fur functioned as a significant contributor in the antibacterial activity of GA (15:1).

### The antimicrobial effect of GA (15:1) involves cross talk with iron homeostasis.

**FIG 5:**
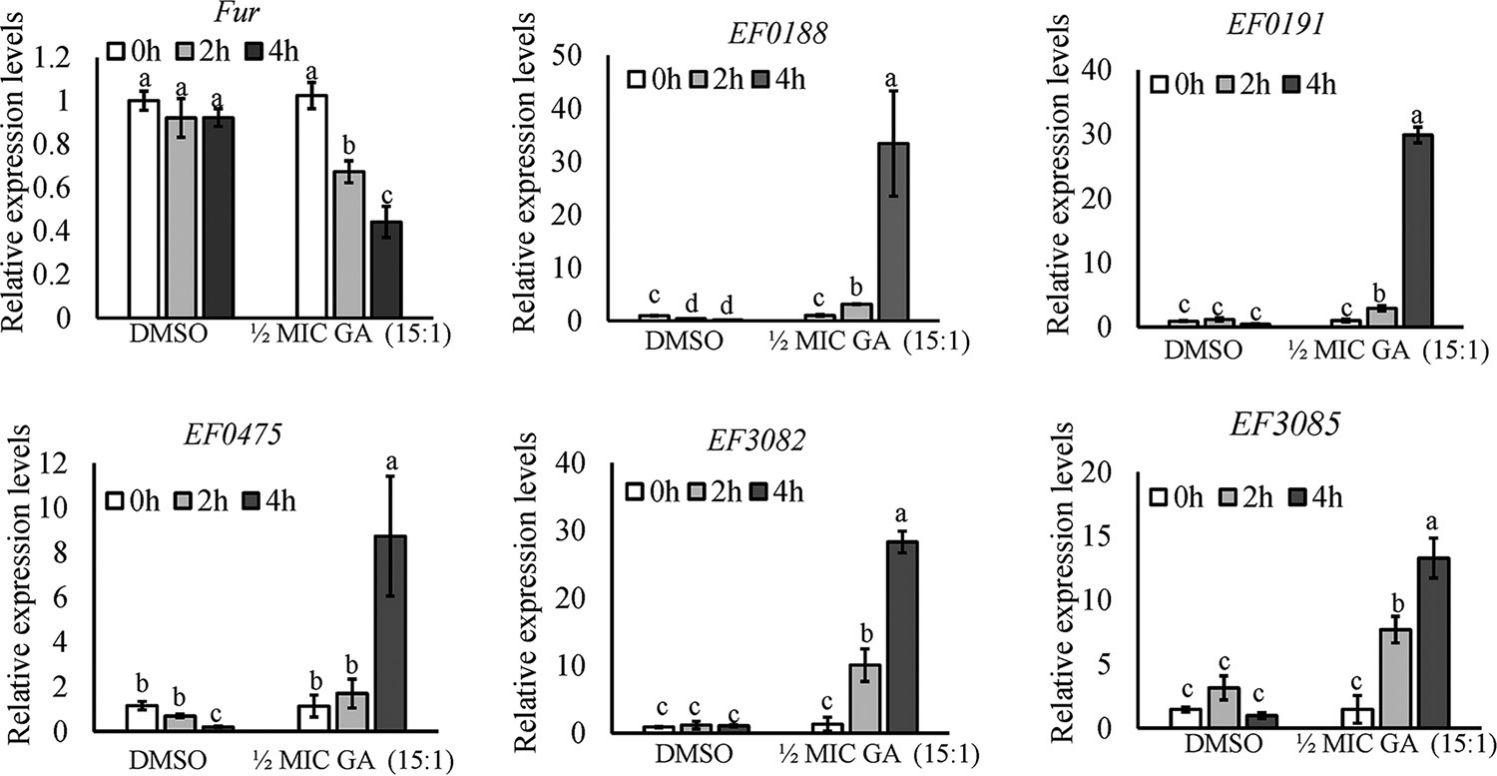
GA (15:1) alters the transcript levels of *fur* and Fur-regulated operons in E. faecalis. qRT-PCR was used to determine the expression levels of *fur*, *EF0188*, *EF0191*, *EF0475*, *EF3082*, and *EF3085* in E. faecalis strain OG1RF at different time points after treatment with 1/2× MIC of GA (15:1). *gdh* was used as an internal control. Data are mean values ± SD from three technical replicates. Different letters indicate statistically significant differences based on ANOVA (*P* < 0.05).

Fur plays a critical role in controlling iron homeostasis in bacterial organisms through regulating the expression of genes involved in iron metabolism (19). To further validate the correlation of iron homeostasis and GA (15:1) susceptibility, the transcript levels of *fur* at 2 h and 4 h when cells were treated with 1/2× MIC of GA (15:1) were measured in E. faecalis strain OG1RF cells by quantitative real-time PCR using *gdh* as an internal control. As shown by the results in Fig. 5, compared with that in the control group, the expression level of *fur* decreased gradually after treatment with 1/2× MIC GA (15:1) in strain OG1RF. In line with that, the transcriptional levels of five Fur-regulated genes (*EF0188*, *EF0191*, *EF475*, *EF3082*, and *EF3085*) involved in iron uptake were significantly increased in the group with GA (15:1) exposure. Furthermore, 2,2′-dipyridyl is a well-known iron chelator, and MIC tests in the presence of GA (15:1) could be performed under iron deprivation by 2,2′-dipyridyl exposure. Our data indicated that the MIC values of the E. faecalis strains in the presence of 500 μM 2,2′-dipyridyl decreased from 2 μg/mL or 4 μg/mL to ≤0.25 μg/mL (Table 2), indicating that iron-deprived bacterial cells had become hypersensitive to GA (15:1) in both clinical isolates and Fur overexpression strains. Of note, 2,2′-dipyridyl exposure resulted in 16-fold and 32-fold decreases of the MIC values of GA (15:1) in the GA (15:1)-induced nonsusceptible isolates (16C51T1 and 16C166T1). Taken together, these results support the participation of Fur and iron homeostasis in the antimicrobial activity of GA (15:1).

**TABLE 2:** MICs of GA (15:1) in E. faecalis strains under iron starvation after treatment with iron chelator 2,2′-dipyridyl

Strain	MIC (μg/mL) of GA (15:1):	
	Without 2,2′-dipyridyl	With 500 μM 2,2′-dipyridyl
OG1RF	4	0.25
OG1RF-pIB166	4	0.25
OG1RF-pIB166-*fur*-1	2	0.25
OG1RF-pIB166-*fur*-2	2	0.25
16C1	4	0.25
16C2	4	0.25
16C41	4	0.25
16C44	4	0.25
16C49	4	0.25
16C50	4	0.25
16C51	4	0.25
16C51T1	32	2
16C166	4	0.25
16C166T1	32	1

**FIG 6:**
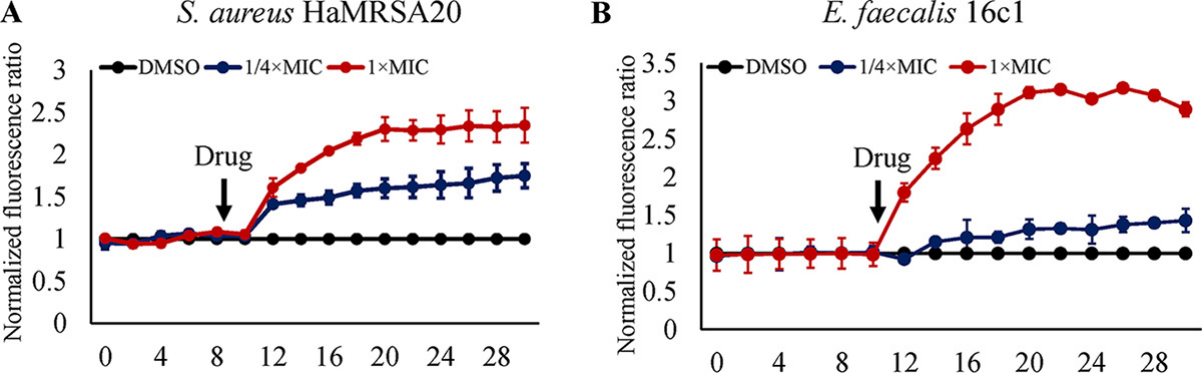
GA (15:1) rapidly dissipated the proton motive force of S. aureus and E. faecalis cells. S. aureus HaMRSA20 (A) and E. faecalis 16C1 (B) cells were treated with DiBAC_4_(3) for 10 min and then treated with 1/4× or 1× MIC GA (15:1) for 20 min. The fluorescence of DiBAC_4_(3) was excited at 492 nm with an emission at 518 nm. Data were normalized to the values of the DMSO-treated control cells.

Iron-sulphur (Fe/S) clusters are ubiquitous cofactors crucial for many biological processes in bacteria. For instance, nine Fe/S clusters in bacteria are integrated into respiratory complex I, which couples NADH oxidation to proton translocation, making iron homeostasis crucial for generating proton motive force (PMF) (20). The components of PMF include membrane potential and the transmembrane proton gradient. The destructive effect of GA (15:1) on the PMF was monitored by using the membrane potential-sensitive fluorescent probe DiBAC_4_(3) {[Bis-(1,3-dibutylbarbituric acid)trimethine oxonol]}. Under normal conditions, DiBAC_4_(3) accumulates in cells with polarized membrane potential. When the membrane potential is perturbed or the membrane permeability is disrupted, an intracellular increase in the fluorescence of DiBAC_4_(3) will be found (21). As shown by the results in Fig. 6, the addition of GA (15:1) led to a rapid elevation of the fluorescence intensity of DiBAC_4_(3), indicating that the cell membrane was depolarized and the membrane PMF was decreased under GA (15:1) exposure.

### Global proteomic response of E. faecalis under GA (15:1) exposure.

**FIG 7:**
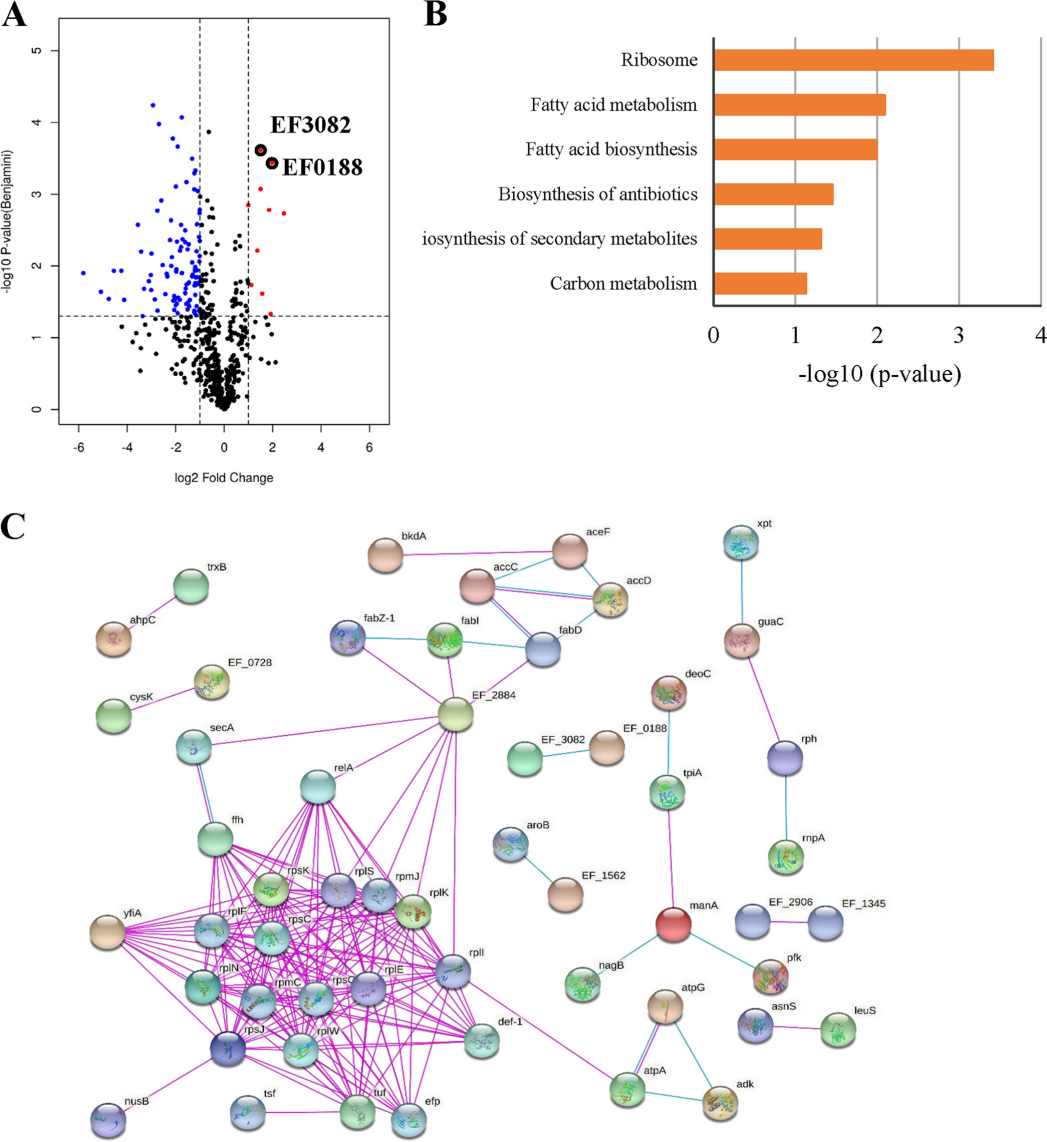
Differential expression of proteins between the control groups and GA (15:1)-treated groups. (A) Volcano plots show log_2_ fold changes of protein levels after treatment of E. faecalis OG1RF cells with GA (15:1) (2 μg/mL, 1/2× MIC) compared to DMSO treatment. Blue dots represent proteins whose expression has been found to be inhibited by GA (15:1). Red dots represent proteins that are upregulated by GA (15:1). Black circles represent ABC transporters of iron. Data represent average values, and *P* values were calculated using a 2-sided 2-sample *t* test; *n* = 3 independent experiments per group. (B) KEGG Pathway terms of the differentially expressed proteins between the two groups. (C) Protein-protein interaction network analysis for proteins differentially expressed between the control groups and GA (15:1)-treated groups. Each node represents a protein, and each edge represents an interaction between proteins. Only known interactions were included. Disconnected nodes are hidden.

Quantitative label-free proteomic analysis was performed to understand the impact of GA (15:1) on E. faecalis. The proteomic responses of E. faecalis strain OG1RF treated with either 1/2× MIC GA (15:1) or dimethyl sulfoxide (DMSO) alone for 2 h were analyzed by mass spectrometry during the exponential growth phase. Overall, 1,228 proteins were confidently identified (matched peptides, ≥1, and false discovery rate [FDR], <0.01), with 10,911 unique peptides quantified. Among the 1,228 proteins quantified, 109 proteins showed significantly different expression levels (≥|2|-fold change, *P* ≤ 0.05) compared with their levels in the control, including 10 upregulated and 99 downregulated proteins after GA (15:1) treatment (Fig. 7A). Detailed information on proteins with significantly different expression levels has been listed in Table S2, using the UniProt database for the expression levels of several categories, such as ribosome, fatty acid biosynthesis and metabolism, energy metabolism, and ATP biosynthesis. The results showed that significant changes might be caused by GA (15:1) treatment (Fig. 7B). Protein-protein interaction (PPI) network analyses were constructed using the STRING database. Consistent with the KEGG Pathway term results, the top hub proteins with the highest degrees of connectivity in the PPI network were enriched in ribosome and protein synthesis functions (Fig. 7C).

Among the differentially expressed proteins, 11 structural components of ribosome-related proteins, including 50S ribosomal proteins L5, L6, L9, L11, L14, L19, L23, and L29, ribosomal protein S3, and 30S ribosomal proteins S10 and S11, were downregulated when E. faecalis was treated with GA (15:1). Ribosomal subunit interface protein YfiA, which is associated with ribosome dimerization (22), RimM, which is associated with the assembly of ribosomal protein S19 into the 30S ribosomal subunit during ribosome maturation (23), ribosome-binding factor A (RbfA) (24), and 50S ribosomal subunit assembly factor BipA, were also downregulated with GA (15:1) treatment. In addition to the canonical ribosome structural proteins, there were decreased amounts of enzymes involved in aminoacyl-tRNA biosynthesis and tRNA modification processes, such as asparagine-tRNA ligase, histidine-tRNA ligase, leucine-tRNA ligase, tyrosine-tRNA ligase 1, and tRNA uridine(34) hydroxylase. Translation elongation factors (EFs) play essential roles during the elongation stage of protein synthesis. In prokaryotes, four EFs are directly responsible for this function (EF-Tu, EF-Ts, EF-G, and EF-P) (25). Three translation elongation factors, including EF-Tu, EF-Ts, and EF-P, were found to be significantly downregulated when E. faecalis was treated with GA (15:1). Moreover, peptide deformylase 1 (Def1), which catalyzes the removal of the *N*-formyl methionine group from nascent polypeptides, an essential step in bacterial protein maturation (26), signal recognition particle protein (Ffh), which is associated with nascent membrane proteins targeting the cytoplasmic membrane, and RNase PH (Rph), which participates in the 3′ maturation of pre-tRNAs and the degradation of rRNA (27), were all downregulated when E. faecalis was exposed to GA (15:1). These results suggested that bacterial protein synthesis was inhibited by GA (15:1), resulting in the inhibition of bacterial growth or death. It is worth noting that two ABC transporters of iron compounds (EF0188 and EF3082) were both upregulated (Fig. 7A), supporting that the iron homeostasis was disrupted with GA (15:1) exposure and the bacterium was maintaining the stability of iron levels through increased uptake of iron.

## DISCUSSION

Due to their ubiquity and ability to survive in extreme environments, multidrug-resistant E. faecalis and S. aureus have become two of the major causes of nosocomial and community-acquired infections. Thus, it is urgent to explore innovative and highly efficient antibacterial agents with unique antimicrobial modes of action or the ability to overcome the widely reported bacterial resistance obstacles (28). Some extracts and bioactive compounds derived from Ginkgo biloba have been reported to exhibit antibacterial activity and were investigated in standard strains of S. aureus and several Gram-positive bacterial species (17, 18, 29). In accordance with the previously reported studies, we showed robust antimicrobial activity of GA (15:1) against clinical S. aureus and E. faecalis isolates from China, suggesting that the MICs range from 2 μg/mL to 4 μg/mL, which is lower than its cytotoxic dose. In addition, GA (15:1) showed a strong inhibitory effect against planktonic cells of clinical multidrug-resistant bacteria, including MRSA and linezolid-intermediate and -resistant and vancomycin-intermediate E. faecalis isolates. Bactericidal activity is an important determinant factor in predicting the clinical outcome of antimicrobial treatment (30). Here, GA (15:1) seemed to exhibit significantly more effective bactericidal activity than commonly used clinical antibiotics like ampicillin and vancomycin. To a large extent, microbes’ biofilm formation is considered an important virulence factor that often results in chronic infections. GA (15:1) also showed potent inhibitory activity against biofilm formation of S. aureus and E. faecalis. More importantly, GA (15:1) could penetrate the mature biofilms and gain access to kill microbial cells embedded in the mature biofilm, suggesting the prospect of its broad application in combination with other drugs to eradicate mature biofilm. These results highlight the potential antimicrobial application of GA (15:1) in the antimicrobial treatment of clinical multidrug-resistant Gram-positive-pathogen infections.

Depending on the redox state, iron exists in a ferrous (Fe^2+^) or ferric (Fe^3+^) form. Iron-related pathways play crucial roles in many redox-sensing proteins as essential cofactors in diverse cellular metabolic pathways, including biosynthesis, respiration, and DNA replication. However, excess iron in bacteria can generate toxic reactive oxygen species that damage biological molecules of bacterial cells (31). Hence, bacteria must develop exquisite iron acquisition strategies and maintain precise iron homeostasis (32, 33). In most bacterial species, the iron-sensing transcriptional regulator Fur mediates iron acquisition and storage (34). Fur is a transcriptional factor that represses iron uptake systems when the iron is plentiful in bacteria. Under iron depletion conditions, Fur-mediated repression becomes inactive and the transcription of iron uptake genes is activated to recover homeostatic iron levels (35). In E. faecalis, four iron uptake system operons (*EF0188*, *EF0191-EF0193*, *EF0475-EF0476*, and *EF3082-EF3085*) are regulated by Fur (19). Here, the whole-genome sequencing of the GA (15:1)-induced nonsusceptible isolates suggested that genetic mutation of the *fur* gene participated in the occurrence of GA (15:1) resistance and that overexpression of Fur might have led to the reduced MICs of GA (15:1), suggesting that Fur might be the target site of GA (15:1). Previous reports have shown that Fur mutation resulted in high levels of intracellular iron accumulation (19, 36). Therefore, we hypothesized that GA (15:1) might destroy the iron homeostasis in bacterial cells and induce an iron starvation response. Our data further demonstrated the upregulated transcriptional levels of *EF0188*, *EF0191*, *EF0475*, *EF3082*, and *EF3085* by quantitative reverse transcription-PCR (qRT-PCR) and the elevated protein levels of EF0188 and EF3082 by proteomic analysis when cells were treated with GA (15:1), supporting the assumption that, due to the depleted intracellular iron and the removal of Fur’s inhibition of the transcription of iron transporter, the iron uptake in E. faecalis was increased when treated with GA (15:1). In particular, iron limitation due to the addition of 2,2′-dipyridyl has also been found to enhance GA (15:1) susceptibility in GA (15:1)-sensitive or induced resistant E. faecalis isolates. However, how iron limitation due to the addition of 2,2′-dipyridyl enhances GA (15:1) activity remains unclear. It might be by increasing the stability or availability of GA in the medium. The details of the underlying mechanisms will be an exciting topic for future research.

Iron was recently found to be associated with biofilm formation, antibiotic susceptibility, and pathogenicity in several bacterial species, although conclusions from multiple studies were highly inconsistent (32, 37–40). In S. aureus, iron restriction enhances biofilm formation, and iron sourced from hemoglobin causes thicker and more structured biofilms than inorganic iron does (41, 42). Mounting numbers of studies have focused on the antimicrobial activity of various antibiotics that were directly or indirectly linked to iron homeostasis. However, in most cases, antibiotic resistance/sensitivity phenotypes were derived from Fur mutants or different chelators. In this study, our data demonstrated that the antibacterial effect of GA (15:1) could be strengthened by the perturbation of iron homeostasis (32, 38). It is also noteworthy that GA (15:1) can disrupt iron homeostasis independently, as shown by the fact that protein and mRNA expression of Fur-regulated iron uptake systems were induced by GA (15:1). Thus, one of the more attractive and feasible uses of GA (15:1) is to explore the application of its combination with other antibiotics that have shown increased sensitivity toward iron starvation.

Mass spectrometry-based quantitative proteomics enlarges our understanding of bacterial behaviors upon different stimuli or environmental conditions. Our current study first described the proteomic response of E. faecalis under GA (15:1) exposure. The results of the proteomic analysis reflected the global view of a direct action of GA (15:1) on E. faecalis and indicated that the differentially expressed proteins were involved in multiple biological functions. The most remarkable finding in our proteomic analysis is the identification of many E. faecalis proteins downregulated by GA (15:1) that are implicated in protein synthesis. The levels of 12 ribosomal protein subunits, ribosome-associated cold shock response protein YfiA, RimM, RbfA, BipA, four aminoacyl-tRNA synthetases, and three vital translation elongation factors, EF-Tu, EF-Ts, and EF-P, were all inhibited by GA (15:1) (Table S1), implying that the protein synthesis machinery is greatly suppressed. Antibiotics that inhibit protein synthesis have been found widely, including macrolides, chloramphenicol, clindamycin, tetracyclines, aminoglycoside, and oxazolidinones (43, 44). Quinolones inhibit bacterial replication by targeting bacterial DNA gyrase (topoisomerase II) and blocking DNA replication (45).

Interestingly, our results showed that iron homeostasis perturbation also induced the transcriptional activation of some iron-related transporters and the zinc operon. These findings suggest the impact of GA (15:1) on iron homeostasis in E. faecalis. Nevertheless, given the fact that the viability of the bacterium was observed to be unaffected or only partially influenced in the *fur* null mutant and the presence of 2,2′-dipyridyl (17, 30, 43), GA (15:1) might execute its roles by other mechanisms of action against Gram-positive bacteria. It is worth noting that GA (15:1) is able to penetrate into the membranes of bacteria and compromise membrane integrity in a concentration-dependent manner, implying that membrane targeting might be another important antibacterial mechanism of GA (15:1).

### Conclusion.

**FIG 8:**
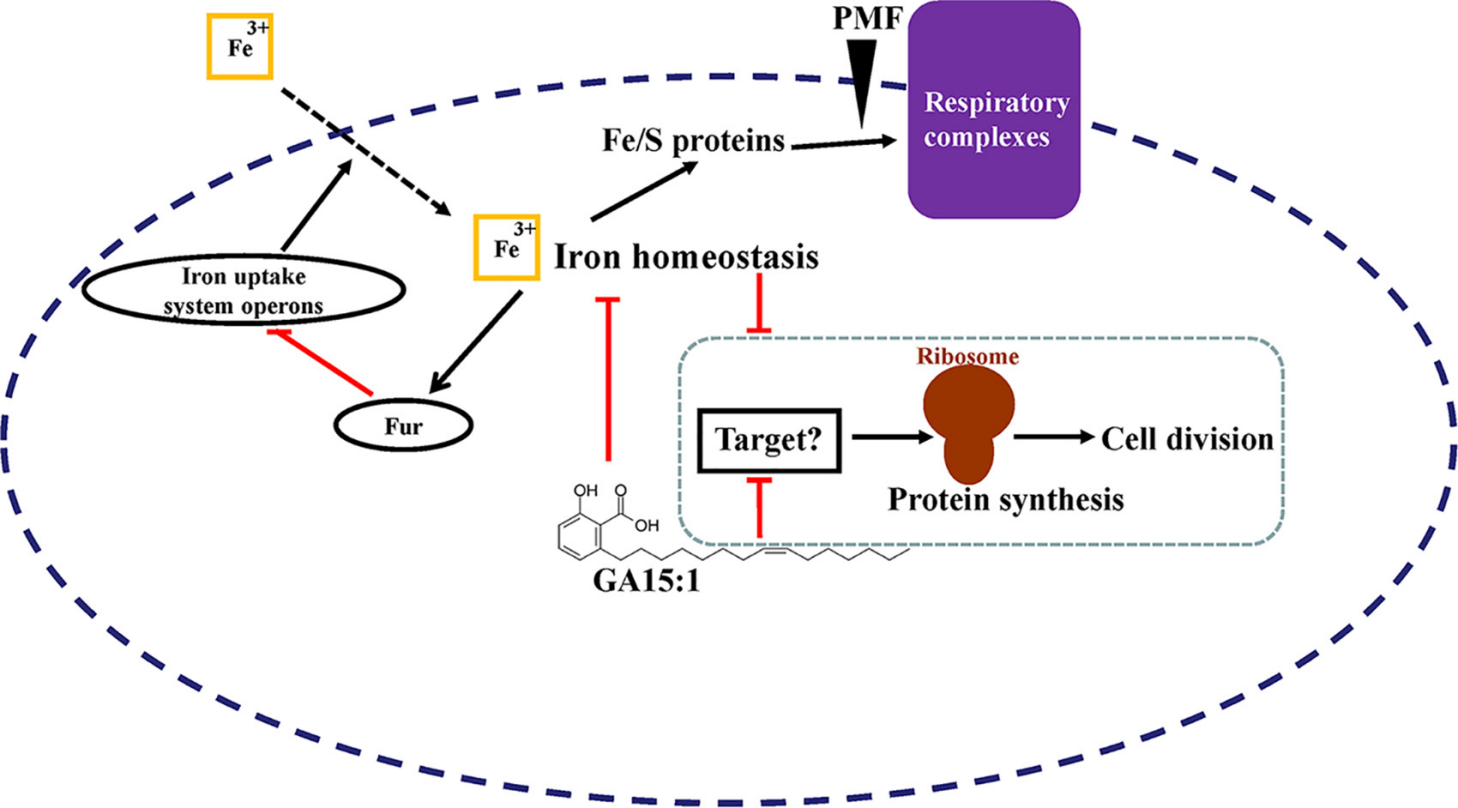
Hypothetical model for the antibacterial mode of action of GA (15:1) against Gram-positive pathogens by cross talk with iron homeostasis. When GA (15:1) is present, it destabilizes the iron homeostasis and creates a state of iron starvation, which triggers the iron starvation response: Fur-mediated repression of iron uptake transporters is relieved, and iron transporters are upregulated, allowing Fe^3+^ to enter the bacterial cells. In addition, GA (15:1) blocks protein biosynthesis and inhibits the growth and cell division of the bacterium through unidentified targets. Meanwhile, the iron starvation condition can strengthen this process.

In the present study, the antimicrobial activities of GA (15:1) against clinical multidrug-resistant S. aureus and E. faecalis isolates *in vitro* and *in vivo* were demonstrated, and GA (15:1) also displayed an impressive ability to inhibit biofilm formation. Moreover, GA (15:1) could penetrate mature biofilm and kill the bacterial cells embedded in the mature biofilm. The antibacterial efficacy of GA (15:1) significantly depended on impairment of iron homeostasis by targeting Fur. The global proteomic response demonstrated that the antibacterial activity of GA (15:1) might be explained by some unidentified target and mechanism, such as the ones affecting protein synthesis (Fig. 8). More investigation is needed to understand the mode of action of GA (15:1).

## MATERIALS AND METHODS

### Bacterial isolates.

E. faecalis and S. aureus clinical isolates employed in this study were retrospectively collected at a Chinese tertiary-care teaching hospital (Nanshan People’s Hospital) and were identified using the Vitek 2 compact system (bioMérieux, Marcy l’Etoile, France), as in our previously reports. Standard strains ATCC 29212 and OG1RF were kept in our laboratory and used as the representative controls in this study. All the isolates were stored in tryptone soy broth (TSB) cultures with 35% glycerol in cryovials at −80°C. Before use, bacterial were subcultured twice in Mueller-Hinton broth for 48 h.

### Drugs and antibiotic susceptibility testing.

GA (15:1) and other commonly used antibiotics were purchased from MedChemExpress (MCE, Shanghai, China). The drug compounds were dissolved in the DMSO to a concentration of 20 mM and stored at −80°C until analyzed. Susceptibility to GA (15:1) and linezolid was determined by the broth dilution method. Briefly, overnight bacterial cultures were adjusted to a turbidity of 0.5 McFarland standard (corresponding to 10^8^ CFU/ml), following by diluting the adjusted inoculum suspensions 1:200 in cation-adjusted Mueller-Hinton broth (CAMHB) that included 2-fold serial dilutions of antibiotics. Then, the microdilution plate was incubated at 37°C for 16 to 20 h. The MIC was defined as the lowest concentration of antibiotics that inhibited the visible growth of bacteria. CAMHB without antibiotic served as the control. MIC values of tetracycline, ampicillin, ciprofloxacin, rifampicin, erythromycin, and nitrofurantoin were obtained using the Vitek 2 compact system. The MIC results of antibiotics, including tetracycline, ampicillin, ciprofloxacin, rifampicin, erythromycin, nitrofurantoin, and linezolid, were interpreted according to Clinical and Laboratory Standards Institute (CLSI) breakpoints (46).

### Proton motive force and membrane permeability assays.

The membrane proton motive force assay was conducted using the membrane potential-sensitive fluorescent probe DiBAC_4_(3). E. faecalis strain OG1RF cells in exponential-growth phase were collected by centrifugation, and the pellets were washed thoroughly three times with HEPES buffer (5 mM, pH 7.2) containing 20 mM glucose. Subsequently, the bacterial cells were resuspended to an optical density at 600 nm (OD_600_) of 0.5 and then incubated with 1 μM DiBAC_4_(3) (catalog no. HY-101892; MCE, Shanghai, China) at 37°C in the dark to let the dye load into the cell membrane. After incubation, the cell suspensions were treated with 1/4× or 1× MIC GA (15:1), and solvent DMSO-treated cells were added as a control group. The fluorescence intensities were monitored in black polystyrene microtiter plates every 2 min for 20 min by using the Cytation 5 cell-imaging multimode reader (BioTek, Winooski, VT, USA) at an excitation wavelength of 492 nm and an emission wavelength of 518 nm. Both the excitation and emission slit widths were set at 5 nm.

### Time-kill studies.

The bactericidal activities of GA (15:1) on the planktonic cells of logarithmic phase were determined by time-kill studies. E. faecalis isolate 16C1 and S. aureus MRSA isolate HaMRSA20, each with a GA (15:1) MIC of 4 μg/mL, were selected, and cultures containing a concentration of 1×, 2×, 4×, or 8× MIC GA (15:1) were monitored at exposure times of 0, 2, 6, 12, and 24 h in the time-kill assays. Bacterial cultures in logarithmic-growth phase were serially diluted with Mueller-Hinton broth, and GA (15:1) was added to make the final concentration. Five-microliter aliquots were plated onto Mueller-Hinton agar. The viable cells were assessed by the CFU counts after incubation for 24 h at 37°C.

### Biofilm analysis.

For quantitative analysis of biofilms of E. faecalis and S. aureus clinical isolates, a crystal violet staining assay was performed in a 96-well polystyrene microtiter plate as we previously reported (47). Briefly, overnight cultures were diluted 1:200 in 200 μl TSBG (TSB with 0.5% glucose) supplied with various concentrations of GA (15:1) (1/8×, 1/4×, 1/2×, 1, and 2× MIC). After 24 h of incubation at 37°C, the OD_600_ values were recorded using the Cytation 5. Supernatant was discarded, and the formed biofilms were washed gently three times with deionized PBS to remove planktonic cells. The biofilms were stained with 1% crystal violet for 20 min, followed by adding ethanol-acetone (80:20, vol/vol) to dissociate the crystal violet. The absorbance at 570 nm was measured. Each treatment was performed in triplicate at least three times.

### *In vitro* selection of E. faecalis exhibiting GA (15:1)-induced resistance.

Two parental E. faecalis isolates (16C51 and 16C166) with MICs of 4 μg/mL were subjected to GA (15:1) to induce resistance *in vitro*. The isolates were subcultured serially in medium supplied with increasing GA (15:1) concentrations of 1×, 2×, 4×, and 8× MIC and successively passaged four times at each concentration. Isolates at each concentration were collected and cultured for three continuous generations without GA (15:1) for further studies. Isolates exhibiting GA (15:1)-induced resistance with a MIC of 32 μg/mL were employed to explore genetic variability by whole-genome sequencing.

### Bacterial whole-genome sequencing.

Chromosomal DNA of two GA (15:1)-induced resistant E. faecalis strains, 16C51T1 and 16C166T1, derived from 16C51 and 16C166, respectively, was prepared for whole-genome sequencing. Nextera library construction and whole-genome sequencing were performed on the Illumina HiSeq sequencing platform by Novogene Co. Ltd., (Beijing, China). The sequencing reads were mapped against the E. faecalis 16C51 strain reference genome in bwa mem software (version 0.7.5a) with standard parameters. Single-nucleotide polymorphisms and insertions/deletions of resistant E. faecalis isolates (16C51T1 and 16C166T1) were identified in MUMmer (version 3.23). The sequences were deposited in NCBI with accession number PRJNA722586.

### Sample preparation for quantitative proteomics.

E. faecalis OG1RF cultures at the exponential-growth phase (OD_600_ of 0.5) were supplemented with GA (15:1) to a final concentration that corresponded to 2 μg/mL (1/2× MIC). The sham group was treated with DMSO. The experiment was performed with three biological replicates for each group. The GA (15:1)-supplemented cultures were incubated at 37°C for 2 h on a shaker at 200 rpm. After that, the bacteria were harvested by centrifugation at 5,000 × *g* for 10 min at 4°C. After washing with cold PBS three times, the cell pellets were suspended in radioimmunoprecipitation assay (RIPA) lysis buffer (1% Triton X-100, 1% deoxycholate, 0.1% SDS) with cOmplete protease inhibitor cocktail (catalog no. 05892970001; Roche, Basel, Switzerland). The suspension was then subjected to three rounds of homogenization with glass beads (diameter 0.1 mm) and centrifuged at 12,000 × *g* for 20 min at 4°C, and the supernatants were collected for protein concentration determination and subsequent quantitative proteomics. The Pierce Micro BCA protein assay kit (catalog no. 23227; Thermo Fisher Scientific, MA, USA) was used to determine the protein concentration. One hundred micrograms of extracted protein was reduced with 10 mM dithiothreitol (DTT) (Sigma-Aldrich Co., St. Louis, MO) for 1 h at 70°C, followed by alkylation using 50 mM iodoacetamide (IAA; Sigma-Aldrich) for 15 min at room temperature in the dark. The samples were then desalted and the buffer changed three times with 100 μl 0.5 M ammonium bicarbonate by using Amicon ultra centrifugal filters (10-kDa cutoff; Millipore, Billerica, MA). The proteins were digested with trypsin (Promega, Madison, WI) at a ratio of 1:50 at 37°C overnight. They were then lyophilized and stored at −80°C.

### NanoLC-MS/MS analysis for quantitative proteomics.

For nanoscale liquid chromatography-tandem mass spectrometry (NanoLC-MS/MS), samples were reconstituted in 30 μl of 0.1% formic acid, and 4 μl of each sample was injected onto an LC system consisting of an UltiMate 3000 RSLCnano system and a C_18_ precolumn (100 μm by 20 mm, Acclaim PepMap 100 C_18_, 3 μm), followed by separation using a C_18_ tip column (75 μm by 250 mm, Acclaim PepMap rapid separation LC [RSLC], 2 μm). Mobile phases A and B were composed of 0.1% formic acid and 80% acetonitrile in 0.1% formic acid, respectively. The elution system started with 5% B for the first 5 min, followed by a linear gradient from 5% B to 38% B in the next 85 min and from 38% B to 95% B in the next 2 min, and then maintained at 95% B for another 3 min at a flow rate of 300 nL/min. The column was coupled to a Q Exactive plus mass spectrometer equipped with the nano-spray ionization (NSI) interface. Mass spectrometer 1 (MS1) scans were acquired over a mass range of 300 to 1,500 *m/z* with a resolution of 70,000, and the corresponding MS2 spectra were acquired at a resolution of 17,500, collected for a maximum of 50 ms. All multiply-charged ions were used to trigger MS/MS scans, followed by dynamic exclusion for 30 s. Singly-charged precursor ions and ions of undefinable charged states were excluded from fragmentation.

### Bioinformatics analysis for quantitative proteomics.

Protein identification and quantification were performed using Proteome Discoverer 2.4 with the Sequest HT against the Uniprot proteome of Enterococcus faecalis strain EnGen0311 (TX0635). A 2-fold cutoff value was applied to determine upregulated and downregulated proteins, in addition to a *P* value of less than 0.05 for at least two technical replicates. The differentially expressed proteins were uploaded into the OmicsBean database for GO (Gene Ontology) annotation, including biological process, cellular component, molecular function, and KEGG pathway analysis. The PPI networks were analyzed by using the Web-based tool OmicsBean.

### Mouse model of S. aureus pneumonia.

BALB/c mice (aged 10 to 11 weeks and weighing 18 to 22 g) were used to establish a mouse pneumonia model. Animals were raised at a constant temperature of 25°C under a regular 12/12-h illumination period and supplied food and water randomly. S. aureus USA300 was cultivated in tryptic soy broth overnight at 37°C and 200 rpm, inoculated into new medium, and incubated to reach the exponential phase. The bacteria were then harvested, washed twice, and resuspended in precooled saline (0.9%) to a final concentration of 1 × 10^10^ CFU/ml. GA (15:1) was dissolved in 40% polyethylene glycol 300 (PEG 300) (catalog no. HY-Y0873; MCE, Shanghai, China) containing 10% DMSO and 5% Tween 80 to a final concentration of 5 mg/mL. For lung infection, mice were injected with 1% sodium pentobarbital (10 mg/kg of body weight) intraperitoneally for anesthesia, and then 20 μl of strain USA300 (containing 2 × 10^8^ CFU) or the same volume of saline (0.9%) was slowly inoculated into the tracheas of mice. After instillation, the mice were kept upright for 1 min to ensure the bacteria were uniformly distributed in the lung. All the mice were randomly assigned to three groups (6 mice per group): (i) a control group, (ii) a GA (15:1) group, and (iii) an ampicillin group. Mice in the GA (15:1) group and the ampicillin group were simultaneously injected intraperitoneally with GA (15:1) or ampicillin at a dose of 25 mg/kg of body weight prior to bacterial infection. No mice exhibited obvious abnormal health during the whole experiment, as the bacterial inoculation dose had been proved not reach the lethal dose by a preliminary experiment. At 24 h, the mice were executed by cervical dislocation. Whole blood was collected from the retro-orbital veins using heparinized capillary tubes for biochemical parameter analysis. The blood was centrifuged at 3,000 rpm for 10 min to obtain serum. Serum alanine aminotransferase (ALT), aspartate aminotransferase (AST), lactic dehydrogenase (LDH), and blood urea nitrogen (BUN) were measured according to the manufacturer’s protocols. The AST, ALT, LDH, and BUN kits were purchased from Andy Gene Biotechnology Co., Ltd. (Beijing, China). The right lung was aseptically harvested for bacterial burden studies. Lung tissues were weighed and transferred into 2-ml homogenization tubes with 1 ml cold PBS and 2.8-mm ceramic beads. Then, the lung tissues were immediately homogenized 3 times for 1 min in a high-throughput homogenizer. The homogenized suspension was serially 10-fold diluted with PBS, and 10-μl amounts of appropriate dilutions were inoculated onto tryptone soy agar (TSA) plates in triplicate. After incubation at 37°C for 24 h, the numbers of S. aureus clones were counted. Bacterial burdens in lung homogenates (CFU/g) were calculated based on the weight of the lung tissue used for homogenization.

### Histopathology.

The mouse tissues (including liver, kidneys, and lung) were fixed in 4% paraformaldehyde. The specimens were embedded in paraffin, serial sections of 4 μm thickness were cut for hematoxylin and eosin (H&E) staining, and the slides were observed and recorded under light microscopy.

### Hemolysis assay.

Fresh human red blood cells (RBCs) from donors were washed with PBS and then resuspended at a concentration of 4% in PBS, and 100-μl amounts were added into a round-bottom 96-well polystyrene microtiter plate. Then, 100-μl amounts of serial 2-fold dilutions of GA (15:1) starting at 256 μg/mL were added to the wells, using Triton X-100 as positive controls. Thereafter, the mixture was incubated for 1 h at 37°C with shaking at 60 rpm. After incubation, the 96-well plate was centrifuged at 1,000 × *g* for 3 min and 100 μl of supernatant from each well was transferred into a new 96-well polystyrene microtiter plate. Absorbance was measured at *A*_450_.

### MTT assay.

The cytotoxic effects of GA (15:1) in A549 and HUVEC cell lines were determined by the MTT {[3-(4,5-dimethyl-2-thiazolyl)-2,5-diphenyl-2H-tetrazolium bromide]} assay. Briefly, cells were washed twice with PBS and then incubated with 0.1 ml serum-free medium containing 0.05% MTT. After incubation for 4 h, the culture medium was removed and 0.1 ml of DMSO was added to each well to solubilize the formazan. The plates were shaken gently for 10 min. Absorbance at 570 nm was measured.

### Gene overexpression.

The overexpression assay of *fur* in E. faecalis OG1RF was constructed as we described previously (48). The coding sequence (CDS) of *fur* was cloned and joined to the BamHI and XhoI restriction enzyme cutting sites of overexpression vector pIB166. The positive overexpression clones were selected by chloramphenicol resistance and verified by quantitative reverse transcription-PCR (qRT-PCR). The primers used for plasmid construction and qRT-PCR are listed in Table S3.

### Statistical analysis.

Continuous variables were analyzed by Student’s *t* test and one-way factorial analysis of variance (ANOVA) using SPSS version 17.0 software (SPSS, Inc., Chicago, IL, USA).

### Animals and ethics statement.

All studies were performed in BALB/c mice purchased from Guangdong Medical Laboratory Animal Center. The experimental protocol and animal use plan in this study were approved by the Animal Ethics Committee of Huazhong University of Science and Technology Union Shenzhen Hospital.

### Data availability.

The raw whole-genome sequencing data were posted in the Sequence Read Archive (SRA) database under BioProject accession number PRJNA722586 in NCBI. The raw proteomics data were deposited in the ProteomeXchange Consortium via the PRIDE partner repository with the data set identifier PXD029978.
