# Supplementary material for: The Mechanism of Action of Ginkgolic Acid (15:1) against Gram-Positive Bacteria Involves Cross Talk with Iron Homeostasis
Source: Microbiol Spectr. 2022 Jan 12;10(1):e00991-21. doi: 10.1128/spectrum.00991-21 (PMC8754111; doi:10.1128/spectrum.00991-21)
Supplement: SUPPLEMENTAL FILE 1 — Supplemental material. Download SPECTRUM00991-21_Supp_1_seq10.pdf, PDF file, 0.6 MB [file spectrum00991-21_supp_1_seq10.pdf]

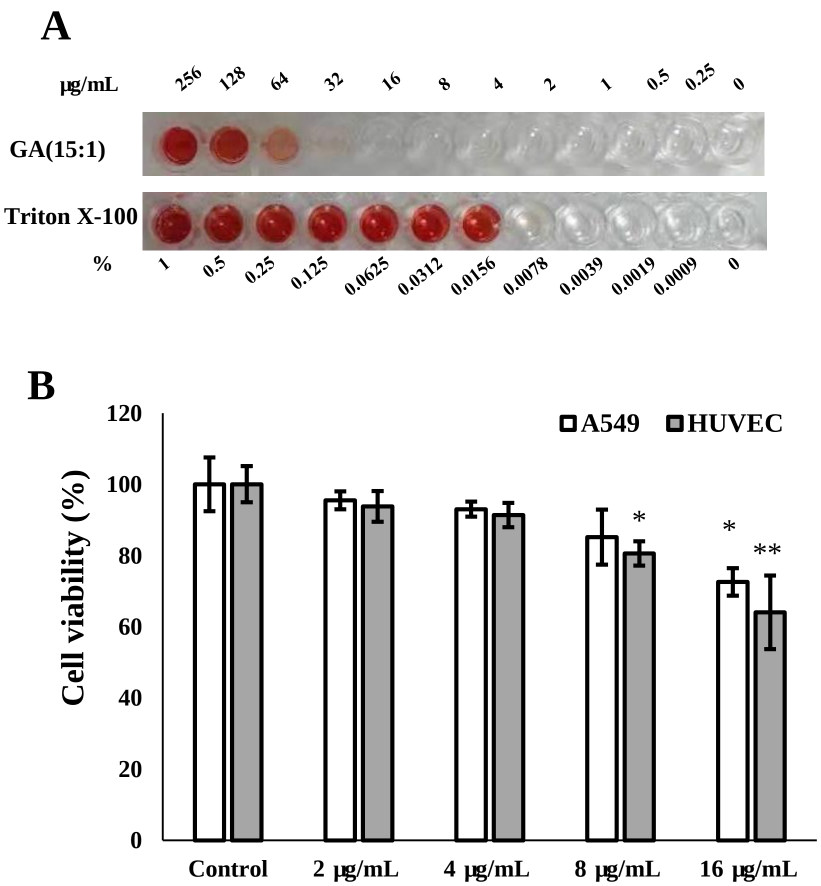

FIG S1 hemolytic activity assay for GA (15:1) against fresh human RBCs and Cytotoxic assay against A549 and HUVEC cell lines.

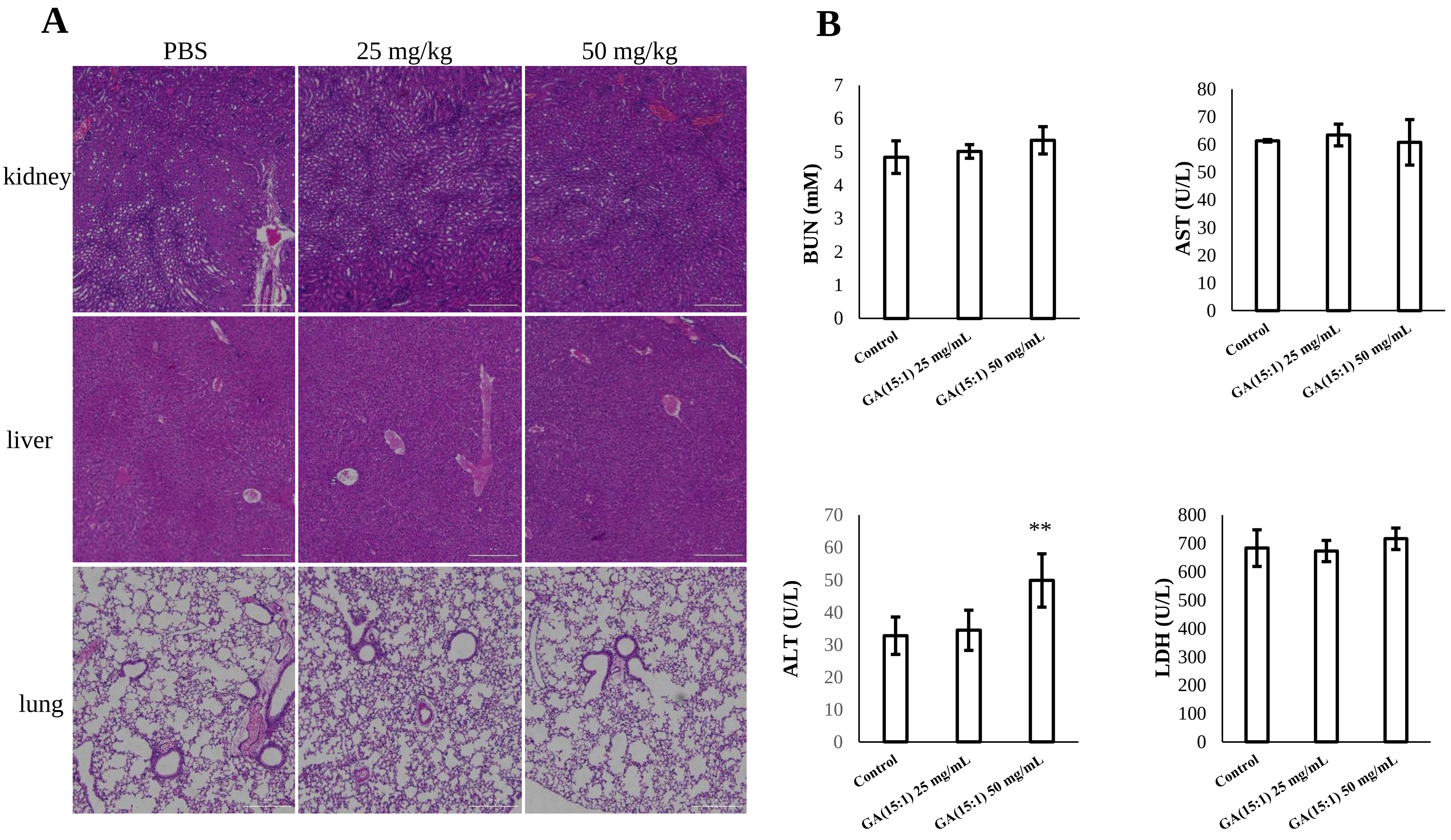

FIG S2 Histopathology of organs and blood parameters collected from mice treated with GA (15:1).

Supplemental Tables S1-S3

Table S1. Mutations in the GA (15:1)-induced non-sensitive isolates detected by the whole-genome sequencing

ref_gene_ID	Nucleotide mutation	Amino acid mutation	description
**16C51T1**			
16C51T1_GM000842	G213T	R71S	MarR family transcriptional regulator
16C51T1_GM001013	A400G	R134G	Fur family transcriptional regulator
16C51T1_GM001052	T848G	D283A	ethanolamine ammonia-lyase light chain family protein
16C51T1_GM000634	A826G	X276Q	Potassium efflux system KefA protein / Small-conductance mechanosensitive channel
16C51T1_GM001288	A449C	K150T	HTH-type dhaKLM operon transcriptional activator dhaS
16C51T1_GM001991	C605T	S202F	sigma-54 factor interaction domain-containing protein
16C51T1_GM002261	T457C	K153E	transcriptional regulator, IclR family
16C51T1_GM002262	T109C	K37E	MerR family transcriptional regulator
16C51T1_GM002375	A65C	E22A	YhgE/Pip C-terminal domain
16C51T1_GM002628	A169G	N57D	RNA-binding protein
**16C166T1**			
16C166T1_GM000207	C41T	A14V	OmpR family two component system sensor histidine kinase VicK/WalK
16C166T1_GM000466	G61A	A21T	Fur family transcriptional regulator
16C166T1_GM000502	C923T	R308Q	Acetaldehyde dehydrogenase, ethanolamine utilization cluster
16C166T1_GM000709	G538A	V180I	Bifunctional (p)ppGpp synthase/hydrolase RelA
16C166T1_GM000786	G434A	R145H	Phage lysin, N-acetylmuramoyl-L-alanine amidase
16C166T1_GM000862	G352A	E118K	general stress protein
16C166T1_GM000864	T59C	V20A	catabolite control protein A
16C166T1_GM000947	G337A	A113T	Dihydrolipoamide acyltransferase component of branched-chain alpha-keto acid dehydrogenase complex
16C166T1_GM001085	G665A	R222Q	ABC transporter ATP-binding protein
16C166T1_GM001139	T731C	H244R	Phosphosugar-binding transcriptional regulator, RpiR family
16C166T1_GM001140	T823G	S275A	6-phosphogluconate dehydrogenase
16C166T1_GM001509	G170A	R57Q	putative membrane spanning protein
16C166T1_GM001404	A452G	D151G	Lsa family ABC-F type ribosomal protection protein
16C166T1_GM001758	C170T	R57Q	hypothetical protein
16C166T1_GM001815	T1022C	Q341R	ABC transporter, ATP-binding protein
16C166T1_GM001658	A109G	S37P	aspartyl/glutamyl-tRNA amidotransferase subunit A
16C166T1_GM002200	A515T	N172I	uracil-DNA glycosylase
16C166T1_GM002110	A55G, A64G	I19V, T22A	YhgE/Pip C-terminal domain
16C166T1_GM002316	G265A	A89T	major facilitator superfamily transporter (MFS) transporter
16C166T1_GM002439	C1501T	G501S	ABC transporter, ATP-binding protein
16C166T1_GM002451	C1649T	S550L	prolyl-tRNA synthetase
16C166T1_GM002533	T110C	X37W	PTS system mannose/fructose/sorbose family IID component
16C166T1_GM002555	A262G	N88D	Methylphosphotriester-DNA--protein-cysteine S-methyltransferase
16C166T1_GM002559	G65A	T22I	conserved hypothetical protein

Table S2. Proteins that showed differential expression following treatment of *E. faecalis* OG1RF with 1/2×MIC GA (15:1).

Accession	Uniprot ID	Gene name	Protein Name	Log2 fold change (GA (15:1)/control)
**Protein synthesis**				
E2YYF3	Q839E1	rpmJ	50S ribosomal protein L36	2.46
E2Z0D8	Q82ZJ1	rpsO	30S ribosomal protein S15	1.37
E2YYG4	Q839F2	rplE	50S ribosomal protein L5	-5.81
E2YYG1	Q839E9	rplF	50S ribosomal protein L6	-3.03
E2YU54	Q839Y6	rplI	50S ribosomal protein L9	-2.75
E2YSK6	Q830Q5	rplK	50S ribosomal protein L11	-4.12
E2YYG6	Q839F4	rplN	50S ribosomal protein L14	-5.08
E2YSZ4	Q833P5	rplS	50S ribosomal protein L19	-2.05
E2YYH4	Q839G2	rplW	50S ribosomal protein L23	-4.55
E2YYG8	Q839F6	rpmC	50S ribosomal protein L29	-1.75
E2YYH0	Q839F8	rpsC	30S ribosomal protein S3	-1.61
E2YYH7	Q839G5	rpsJ	30S ribosomal protein S10	-1.51
E2YYF1	Q839E0	rpsK	30S ribosomal protein S11	-2.75
E2YUG8	Q834A6	yfiA	Ribosome hibernation promoting factor	-2.03
E2YSZ6	Q833P4	rimM	Ribosome maturation factor RimM	-1.04
E2YWP9	Q835U7	rbfA	Ribosome-binding factor A	-2.42
E2YZW1	Q831P1	bipA	50S ribosomal subunit assembly factor BipA	
E2YVU0	Q831X4	asnS	Asparagine--tRNA ligase	-1.49
E2YT60	Q833I1	hisS	Histidine--tRNA ligase	-2.18
E2YZH4	Q837N5	leuS	Leucine--tRNA ligase	-1.03
E2YXL6	P0DM33	tyrS1	Tyrosine--tRNA ligase 1	-1.46
E2YX98	Q837T2	trhO	tRNA uridine(34) hydroxylase	-1.01
E2YWF3	Q839G8	tuf	Elongation factor Tu	-3
E2YZP8	Q831V0	tsf	Elongation factor Ts	-1.19
E2YZA4	Q838Z5	efp	Elongation factor P	-2.16
E2Z0D9	Q82ZJ0	def	Peptide deformylase	-1.72
E2YVH3	Q834F5	ffh	Signal recognition particle protein	-1.41
E2YVN7	Q836I9	rph	RNase PH	-1.55
E2Z014	Q831F9	glyA	Serine hydroxymethyltransferase	-1
E2YVA5	Q834Q6	cysK	Cysteine synthase	-1.74
**Cell division**				
E2YY78	H7C713	divIVA	Cell division protein DivIVA	-2.06
E2YY72	O07111	ftsA	Cell division protein FtsA	-3.36
**DNA replication and transcription**				
E2YY54	Q836W8	nusB	Transcription antitermination protein NusB	-1.15
E2YXV3	Q837G4	polA	DNA polymerase I	-1.02
E2YVU7	Q831Y0	xpt	Xanthine phosphoribosyltransferase	-1.99
E2YY61	Q836W1	recN	DNA repair protein RecN	-1.04
E2YU48	Q839Z1	gyrB	DNA gyrase subunit B	-2
E2YVD2	H7C794	parE	DNA topoisomerase IV subunit B	-1.32
E2YZS8	Q831S1	guaC	GMP reductase	-1.01
E2Z013	Q831G0	upp	Uracil phosphoribosyltransferase	-1.16
**Cell wall**				
E2YWV6	Q835N8	trxB	Thioredoxin reductase	-1.23
E2YSM0	Q830P3	murB	UDP-N-acetylenolpyruvoylglucosamine	-1.19

			reductase	
E2YW24	Q832N2	EF2191	dTDP-4-dehydrorhamnose reductase	-1.91
**Fatty acid biosynthesis and metabolism**				
E2YXN9	Q830B4	accD	Acetyl-coenzyme A carboxylase carboxyl transferase subunit beta	-2.19
E2YXN1	Q830B0	acpP	Acyl carrier protein	-1.46
E2YVA2	Q834J1	bkdA	2-oxoisovalerate dehydrogenase subunit alpha	-1.82
E2YXN8	Q830B3	accC	Biotin carboxylase	-1.55
E2YWX1	Q835M2	aceF	Dihydrolipoamide acetyltransferase component of pyruvate dehydrogenase complex	-1.21
E2YZA7	Q820V3	fabZ1	3-hydroxyacyl-[acyl-carrier-protein] dehydratase FabZ	-1.98
E2YZA9	Q820V5	fabI	Enoyl-[acyl-carrier-protein] reductase [NADH] FabI	-2.54
E2YXN3	Q830B1	fabD	Malonyl CoA-acyl carrier protein transacylase	-4.75
**Energy metabolism and ATP biosynthesis**				
E2YWW8	Q835M5	EF1352	Magnesium-transporting ATPase, P-type 1	-1.51
E2YVA7	P37061	nox	NADH oxidase	-1.78
E2YYY2	P59687	nagB	Glucosamine-6-phosphate deaminase	-1.2
E2YS42	Q831A4	atpG	ATP synthase gamma chain	-1.12
E2YS43	Q831A3	atpA	ATP synthase subunit alpha	-2.34
E2YYF5	Q839E3	adk	Adenylate kinase	-1.32
E2YUG6	Q834A7	secA	Protein translocase subunit SecA	-1.08
E2YT51	Q833J0	tpiA	Triosephosphate isomerase	-1.19
E2YS23	Q831C2	manA	Mannose-6-phosphate isomerase	-1.12
E2YYC1	Q836R3	pfkA	ATP-dependent 6-phosphofructokinase	-2.37
**ABC transporter**				
E2YWE2	Q839H9	EF0188	Iron compound ABC transporter	1.98
E2YTN1	Q82ZZ1	EF2906	ABC-type sugar transport system	1.92
E2YXW8	Q837E9	EF0893	Amino acid ABC transporter	1.86
E2YS70	Q830X6	EF2642	Glycine betaine/L-proline ABC transporter	1.57
E2Z0F4	Q82ZH5	EF3082	Iron compound ABC transporter	1.51
E2YWW3	Q835N2	EF1345	Sugar ABC transporter	-1.61
E2YWJ8	Q835Z7	EF1221	Spermidine/putrescine ABC transporter	-1.77
E2YXU1	Q837H4	EF0865	Glycine betaine/carnitine/choline transporter	-1.9
E2YVZ3	Q832K5	EF2221	ABC transporter	-2.59
**Others**				
E2YXT4	Q837I1	pip	Phage infection protein	1.51
E2YXJ0	Q838B1	EF0661	Oligoendopeptidase F, putative	1.12
E2YU41	Q82YV0	rnpA	Ribonuclease P protein component	1
E2YSI9	Q830S4	EF2698	Tellurite resistance protein, putative	-1
E2YVQ9	Q836G7	EF1145	Uncharacterized protein	-1.06
E2YT13	Q833M6	EF1918	Uncharacterized protein	-1.09
E2YY01	Q837B7	EF0926	DNA-binding response regulator	-1.13
E2YWD3	Q839I8	EF0177	Basic membrane protein family	-1.14
E2YVG1	Q834K3	hslV	ATP-dependent protease subunit HslV	-1.16
E2YSS8	Q82ZW5	EF2932	AhpC/TSA family protein	-1.19
E2YS72	Q830X4	EF2644	Diacylglycerol kinase catalytic domain protein	-1.21
E2YXA8	Q837T8	EF0742	Uncharacterized protein	-1.22
E2YXC1	Q837V0	EF0728	Uncharacterized RNA methyltransferase EF_0728	-1.22

E2YWV6	Q835N8	trxB	Thioredoxin reductase	-1.23
E2Z0X2	Q82Z22	EF3257	Oxidoreductase, pyridine nucleotide-disulfide family	-1.23
E2YVQ6	Q836H0	EF1142	Hydrolase, haloacid dehalogenase-like family	-1.3
E2YT63	Q833H8	relA	(p)ppGpp synthase	-1.32
E2YS71	Q830X5	EF2643	Uncharacterized protein	-1.44
E2YW81	Q93K67	arcA	Arginine deiminase	-1.45
E2YS97	Q830V2	cutC	Copper homeostasis protein CutC	-1.56
E2YUC9	P36922	EF1730	Putative Cys-tRNA(Pro)/Cys-tRNA(Cys) deacylase EbsC	-1.59
E2YW03	Q832L4	EF2212	Uncharacterized protein	-1.6
E2YSM6	H7C7A0	ahpC	Alkyl hydroperoxide reductase C	-1.75
E2YV73	Q834T0	EF1553	TPR domain protein	-1.83
E2YYI8	Q839B7	EF0259	S4 RNA-binding domain protein	-1.91
E2YZV6	Q831P5	EF2455	CAP_assoc_N domain-containing protein	-1.95
E2YVD3	Q834M9	EF1616	CoA-binding domain protein	-1.95
E2YZR4	Q831T4	EF2415	Uncharacterized protein	-1.97
E2YZD5	Q837P0	EF0794	Uncharacterized protein	-2.1
E2YWD0	Q839J1	deoC	Deoxyribose-phosphate aldolase	-2.12
E2YTN8	Q82ZZ9	EF2898	Peptidyl-prolyl cis-trans isomerase	-2.23
E2YV83	H7C796	EF1562	Phospho-2-dehydro-3-deoxyheptonate aldolase, putative	-2.69
E2YYZ5	Q838J4	EF0453	OsmC/Ohr family protein	-2.86
E2YV71	Q834T3	hup	DNA-binding protein HU	-2.93
E2YUE5	Q834C4	EF1744	General stress protein, putative	-3.01
E2YV84	P0DH71	aroB	3-dehydroquinate synthase	-3.1
E2YUF4	Q834B6	EF1753	DUF4097 domain-containing protein	-3.3
E2YRR2	Q836D5	EF1180	UPF0337 protein EF_1180	-3.42
E2YXG8	Q837Z3	EF0681	UPF0342 protein EF_0681	-3.55
E2YTL1	Q82ZX2	EF2925	Cold-shock domain family protein	-4.24

Table S3. Sequence of primers used in this study.

Primers	Sequence (5' to 3')	Purpose
Fur-sense	AAGAAGCCAATATCCGAATTAC	*quantitative RT-PCR*
Fur-antisense	AACCGTTGCTACACTCAT	
gdh-sense	CGTGATGAACCGAATGTG	
gdh-antisense	GTTAATGCGTGTGCCTTT	
EF0188-sense	CACATGAAGTAACAGATACCTTAG	
EF0188-antisense	CTTGAATGCTGCCTTGTC	
EF0191-sense	ATGGTCGTCAAGTGATTAGT	
EF0191-antisense	AATGTTGAAGTTCTTGGTTCTT	
EF045-sense	TGGACATGGGCTTAACAA	
EF045-antisense	CAAGATTAGTTCCGCTTCAC	
EF3082-sense	ATGGGCAAAGTGTTTCTTAC	
EF3082-antisense	ACATCTGGTTGAAGCATAATG	
EF3085-sense	TTGCTGGTAGTACGATGAG	
EF3085-antisense	AATTGCCGTTCGATAGAGA	
Fur-F	GGATCCatggacaacgtattggttaaaaatg	Generation of pIB166-*Fur*
Fur-R	CTCGAGttacacattctcctgttgtgc	
